# Insights into Enzyme Reactions with Redox Cofactors in Biological Conversion of CO_2_

**DOI:** 10.4014/jmb.2306.06005

**Published:** 2023-06-26

**Authors:** Du-Kyeong Kang, Seung-Hwa Kim, Jung-Hoon Sohn, Bong Hyun Sung

**Affiliations:** 1 Synthetic Biology Research Center, Korea Research Institute of Bioscience and Biotechnology (KRIBB), Daejeon 34141, Republic of Korea; 2 Department of Biosystems and Bioengineering, Korea University of Science and Technology (UST), Daejeon 34113, Republic of Korea

**Keywords:** CO_2_ assimilation, CO_2_-fixation pathway, C1 reduction, redox cofactor, synthetic biology

## Abstract

Carbon dioxide (CO_2_) is the most abundant component of greenhouse gases (GHGs) and directly creates environmental issues such as global warming and climate change. Carbon capture and storage have been proposed mainly to solve the problem of increasing CO_2_ concentration in the atmosphere; however, more emphasis has recently been placed on its use. Among the many methods of using CO_2_, one of the key environmentally friendly technologies involves biologically converting CO_2_ into other organic substances such as biofuels, chemicals, and biomass via various metabolic pathways. Although an efficient biocatalyst for industrial applications has not yet been developed, biological CO_2_ conversion is the needed direction. To this end, this review briefly summarizes seven known natural CO_2_ fixation pathways according to carbon number and describes recent studies in which natural CO_2_ assimilation systems have been applied to heterogeneous in vivo and in vitro systems. In addition, studies on the production of methanol through the reduction of CO_2_ are introduced. The importance of redox cofactors, which are often overlooked in the CO_2_ assimilation reaction by enzymes, is presented; methods for their recycling are proposed. Although more research is needed, biological CO_2_ conversion will play an important role in reducing GHG emissions and producing useful substances in terms of resource cycling.

## Introduction

Net-zero carbon emission is a worldwide task owing to the rapidly increasing greenhouse gas (GHG) levels in the atmosphere. Among the human-made GHGs, C1 molecules, especially carbon dioxide (CO_2_), are receiving considerable attention as they account for almost 70% of the GHGs that are contributing to the rapidly intensifying global warming and climate change [1]. The CO_2_ concentration in the atmosphere has markedly increased since the Industrial Revolution and is now almost 50% higher than preindustrial levels, at 412 parts per million (ppm) on average. Global surface temperatures have increased, in correlation with atmospheric concentrations of CO_2_, by nearly 1°C compared to preindustrial levels [2, 3]. This increase of 1°C has caused a number of distinct changes, including higher temperatures on land and in the oceans, glacier melting, and increased frequency and severity of precipitation or drought. Furthermore, many experts have predicted that these changes will become more severe with a global warming of 1.5°C over preindustrial levels and that the damages will be difficult to reverse at 2°C of global warming [4]. In addition to climate change, rising atmospheric concentrations of GHGs have the potential to threaten ecosystems and eventually affect humans adversely [5-7]. Therefore, to maintain global temperatures below 1.5°C, efforts to achieve a phased goal of reducing carbon emissions by 45% from 2005 to 2030 and eventually reaching net-zero carbon emissions by 2050 are desperately needed [8]. Solving the problem of the CO_2_ in the atmosphere is not only an environmental issue but also provides the opportunity to use a substrate on which the carbon skeleton of profitable materials, such as fuels and various chemicals, can be built. Therefore, more attention is now being paid to CO_2_ sequestration with reference to carbon capture, utilization, and storage (CCUS) than to carbon capture and storage (CCS). Carbon storage technology involves capturing the carbon in the atmosphere and transporting carbon gases underground, typically using geological space as a carbon storage reservoir. Studies on CCS technology using industrial solid waste and steel-making slags, as carbon storage, are ongoing [9-11]. CCUS technology involves carbon capture and utilization to generate value-added products through subsequent reactions [12]. Biological CO_2_ reduction or assimilation, which will be introduced in this paper, is included in the CCUS in terms of generating biomass and valuable materials.

Studies on the mitigation and use of CO_2_ are being conducted in various applications such as metal- and nanomaterials-fused electrochemical catalysis, photocatalysis, and biological catalysis [13-15]. Among these, biological CO_2_ conversion is an environmentally friendly and highly substrate-specific and reusable method that recycles CO_2_ substrates into value-added products. Ecosystems can efficiently reduce CO_2_ emissions through biological CO_2_ assimilation, which can be performed by plants and microorganisms. However, the large amounts of CO_2_ gases emitted by human activities have already exceeded the assimilation capacity of the natural ecosystem, causing excessive global warming [16]. To increase the carbon fixation efficiency beyond that of natural cycles, novel biotechnologies, such as synthetic biology, need to be incorporated into natural biological carbon reduction systems. Therefore, biomimetic strategies such as rebuilding paths by introducing partial heterologous carbon fixation pathways into in vivo and in vitro models may be crucial in solving the carbon fixation problem. In this review, we briefly provide an overview of the natural CO_2_ fixation pathways with enzymes and cofactors and introduce the application of biological CO_2_ assimilation studies. In addition, we discuss the cofactors called redox partners, which are essential components that play important roles in regulating C1 fixation. Our aim with this review was to provide a better understanding of the overall biological CO_2_ fixation pathways in nature, including not only C1-converting enzymes but also their important redox cofactors in CO_2_ reduction, and to represent novel possibilities for biological C1 fixation.

## CO_2_ Fixation Pathways in Nature

**Fig. 1:**
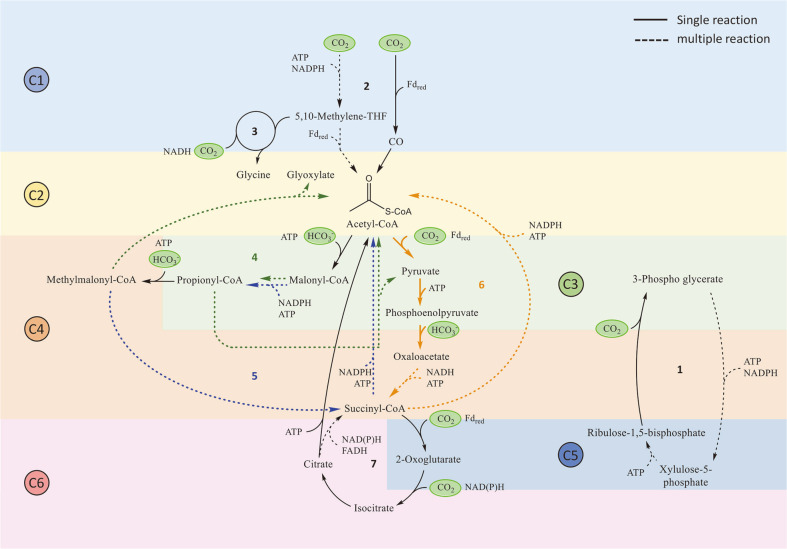
An overview illustration of major CO_2_ assimilation pathways in nature. (1) Calvin-Benson-Basham cycle; (2) Wood-ljungdahl pathway; (3) reductive glycine pathway; (4) 3-hydroxypropionate bicycle; (5) 3-hydroxypropionate/4-hydroxybutyrate cycle; (6) dicarboxylate/4-hydroxybutyrate cycle; and (7) reductive citric acid cycle. The 3-HP bicycle, 3-HP/ 4-HB cycle, and DC/4-HB cycle are represented by green, blue, and orange lines, respectively.

To date, seven carbon fixation pathways have been identified in nature. Each pathway can assimilate different types of C1, such as gaseous CO_2_ and bicarbonate (HCO_3_^-^). The Calvin–Benson–Basham (CBB), Wood–Ljungdahl pathway (WLP), reductive glycine pathway (rGlyP), and reductive tricarboxylic acid cycle (rTCA) can fix gaseous CO_2_, whereas 3-hydroxypropionate (3-HP) bicycle and 3-hydroxypropionate/4-hydroxybutyrate (3-HP/4-HB) can fix bicarbonate. Both forms of carbon can be assimilated in the dicarboxylate/4-hydroxybutyrate (DC/4-HB) cycle. All pathways except the CBB cycle involve acetyl-CoA. A comprehensive representation of all natural CO_2_ fixation pathways is depicted in Fig. 1 based on the carbon number. The carbon-fixing enzymes and cofactors are listed in Table 1, along with their carbon-assimilating reaction and simplified change in carbon number.

The CBB cycle, also known as the reductive pentose phosphate cycle, is the predominant carbon fixation pathway in plants and photosynthetic bacteria. In this cycle, CO_2_ and water are converted into organic compounds using cofactors such as light-driven ATP and NADPH [17]. The key enzyme used for CO_2_ fixation in the CBB cycle is ribulose-1,5-bisphosphate carboxylase/oxygenase (RuBisCO), which is categorized as a lyase. RuBisCO catalyzes the addition of CO_2_ to ribulose 1,5-bisphosphate (C5) and splits into two molecules of 3-phosphoglycerate (C3).

The WLP is one of the noncyclic pathways among the seven natural carbon fixation pathways and is also denoted as the reductive acetyl-CoA route. In this pathway, CO_2_ molecules are reduced into formate and carbon monoxide (CO) via formate dehydrogenase (FDH) and CO dehydrogenase in the initial stage, respectively. In this pathway, two molecules of CO_2_ are converted into one molecule of acetyl-CoA (C2) using cofactors such as NADPH, ATP, and reduced ferredoxin (Fd_red_) [18].

The rGlyP is a CO_2_ fixation metabolic pathway found in anaerobic bacteria, eukaryotes, and plants [19-21]. The initial reaction in this pathway starts with reducing CO_2_ to formate or directly reducing formate to 10-formyltetrahydrofolate (10-formyl-THF) [22]. The subsequent reactions of 10-formyl-THF produce 5,10-methylene-THF, which is used as a one-carbon unit for attaching additional CO_2_ to produce glycine. This process is catalyzed by a multienzyme complex called the glycine cleavage/synthase system (GCS), consisting of aminomethyltransferase, glycine dehydrogenase, and dihydrolipoyl dehydrogenase [23]. To assimilate CO_2_ into 5,10-methylene-THF to generate glycine (C2), NADH and NH_3_ are required as cofactors [24].

The 3-HP bicycle was discovered in the thermophilic green nonsulfur bacteria, *Chloroflexus aurantiacus*, which obtains energy from light. In this cycle, two molecules of bicarbonate are fixed by acetyl-CoA carboxylase and propionyl-CoA carboxylase, and these enzymes generate C3 and C4 products, respectively, in the presence of ATP. The initial step of the 3-HP cycle is the conversion of acetyl-CoA (C2) to malonyl-CoA (C3) by acetyl-CoA carboxylase; after sequential steps, propionyl-CoA (C3) is converted into methylmalonyl-CoA (C4) by propionyl-CoA carboxylase [25]. In the last step of the 3-HP cycle, malyl-CoA, made from methylmalonyl-CoA, is split into acetyl-CoA and glyoxylate; (s)-citramalyl-CoA, which is formed through several steps after combining glyoxylate and propionyl-CoA, is divided into acetyl-CoA and pyruvate.

The 3-HP/4-HB cycle is a carbon fixation pathway that was discovered in *Sulfolobales* such as *Metallosphaera* and *Thaumarchaeota* [26,27]. The cycle starts with the conversion of acetyl-CoA (C2) into malonyl-CoA (C3) by incorporating bicarbonate; then, following sequential steps, propionyl-CoA (C3) is carboxylated to succinyl-CoA (C4) through methylmalonyl-CoA (C4) by incorporating additional bicarbonate. The key enzyme of inorganic carbon assimilation is acetyl-CoA/propionyl-CoA carboxylase, which generates two acetyl-CoA molecules from one acetyl-CoA, with 3-HP and 4-HB as key intermediates [28]. In this pathway, NADPH and ATP function as essential cofactors for the production of methylmalonyl-CoA from acetyl-CoA.

The DC/4-HB cycle was discovered in the anaerobic hyperthermophilic archaea, *Ignicoccus* species, which use CO_2_ with sulfur and hydrogen for their growth. CO_2_ is assimilated via acetyl-CoA to pyruvate using reduced ferredoxin (Fd_red_) by pyruvate synthase, and bicarbonate is transformed into phosphoenolpyruvate to oxaloacetate by phosphoenolpyruvate carboxylase. The cofactors needed for C1 assimilation in this pathway include reduced Fd, ATP, and NAD(P)H [26].

The rTCA cycle is the reverse of the TCA cycle, so is also called the reverse TCA cycle. In this cycle, two molecules of CO_2_ are formed as C6 products from a C4 material through two steps. The first step involves catalysis by 2-oxoglutarate oxidoreductase, which produces 2-oxoglutarate (C5) from succinyl-CoA (C4) using one molecule of CO_2_ and the reducing energy from Fd_red_. In the second step, isocitrate dehydrogenase provides CO_2_ fixation into 2-oxoglutarate (C5) to generate isocitrate (C6) [29]. Cofactors such as NAD(P)H, ATP, Fd_red_, and FADH are used in rTCA cycle. In addition, the model in which isocitrate lyase is introduced has the shortest pathway to reduce two molecules of CO_2_ per cycle [30, 31]. The key elements of these CO_2_ fixation pathways are heterologously expressed and used in model microorganisms.

## In Vivo Applications of Natural Biological CO_2_ Assimilation Systems

Many in vivo heterologous assimilation studies have been conducted to adapt metabolic pathways to use CO_2_. RuBisCO, which is involved in the CBB cycle, has been extensively studied to generate microorganisms with a non-native CBB cycle for CO_2_ fixation. Hence, diverse approaches have been considered for constructing CO_2_ assimilation bio-platforms using various techniques, such as genetic modification, strain evolution, and computational analyses of metabolic flux. For CO_2_ fixation, *Escherichia coli* was rewired to supply ATP and NADH from pyruvate through the TCA cycle as an energy module, fixing CO_2_ by RuBisCO as an assimilation module [32]. To strengthen the CO_2_ fixation ability of the strain that introduces RuBisCO and phosphoribulokinase, laboratory evolution and computational analyses were conducted, which resulted in an autotroph strain that has the ability to use CO_2_ for biomass under higher CO_2_ concentrations than the ancestral strain [33]. Similar to the two-module system study, autotrophic *E. coli* engineered with RuBisCO and FDH was constructed to convert CO_2_ to all-carbon biomass while regenerating cofactors such as NADH and ATP [34]. In addition to introducing only RuBisCO, researchers have reduced exogenous and endogenous CO_2_ by introducing the CBB operon from *Rhodobacter sphaeroides* and 20 heterologous genes related to CO_2_-concentrating mechanisms into *E. coli* [35, 36]. The ribulose-monophosphate (RuMP) pathway catalyzes the conversion of formaldehyde derived from CO_2_ or methane into biomass. Activating sedoheptulose bisphosphatase in RuMP pathway into *E. coli* led to three-fold-enhanced formaldehyde incorporation ability [37]. In addition, *E. coli* with a reconstructed metabolic pathway into which a RuMP shunt was introduced effectively converted methanol and sarcosine-derived formaldehyde into biomass [38]. RuMP-introduced methylotrophic *E. coli* was developed via flux balance analysis [39]. Furthermore, studies on converting C1 substances such as methanol, formate, and CO_2_ into various products via a modified serine cycle and rGlyP have also been conducted [40-42]. Carbonic anhydrase (CA), an enzyme that can directly convert CO_2_ to the ionic form of bicarbonate (HCO_3_^-^), was used to construct *E. coli* that could produce an industrially attractive material, calcium carbonate (CaCO_3_) [43].

Other bio-platforms for *E. coli* have also been studied for CO_2_ fixation. Cyanobacteria, which are representative photosynthetic marine bacteria, have been extensively studied for their ability to produce various biochemicals and biofuels while fixing CO_2_. RuBisCO in cyanobacteria was used as a CO_2_-fixing module and oleochemical-producing modules were added. A strain overexpressing the efflux pump and deficient *aas* gene coding for acyl-acyl carrier protein (acyl-ACP) demonstrated a high free fatty acid (FFA) content of 640 mg/l [44]. Another mutant introducing thioesterase A and fatty acid photodecarboxylase with acyl-ACP deficient *Synechocystis* sp. produced 111.2 mg/l of fatty alkanes [45]. In addition, *Synechocystis* sp., lacking the acyl-ACP synthetase gene (*Δaas*) and overexpressing the genes *sfp* and *car* encoding phosphopantetheinyl transferase and carboxylic acid reductase, respectively, produced over 905 mg/l of 1-octanol from CO_2_ [46].

## In vitro Studies of Biological CO_2_ Assimilation

Various in vitro enzymatic CO_2_ reductions have been studied by investigating and exploring novel biocatalysts, further engineering wild-type enzymes, optimizing reaction conditions, introducing cascade systems, and immobilizing enzymes to increase C1 assimilation efficiencies. Researchers have produced methanol from CO_2_ through formate and formaldehyde using FDH, formaldehyde dehydrogenase (FalDH), and alcohol dehydrogenase enzymes. The representative CO_2_ reduction enzyme FDH, which produces formate from CO_2_, has been extensively studied. A newly discovered FDH from *Thiobacillus* sp. (TsFDH) has approximately 85-fold higher activity in reducing CO_2_ than the FDH from *Candida boidinii*, which is commercially available but has weak CO_2_ reduction activity [47]. Following this study, other FDHs have continuously been discovered from new species, such as *Candida methylica*, *Chaetomium thermophilum*, and *Rhodococcus jostii*, and examined for CO_2_ reduction [48, 49]. In addition, the introduction of a multienzyme cascade reaction with a cofactor regeneration system and the optimization of C1 reduction conditions using novel FalDH from *Burkholderia multivorans* showed up to 500-fold increased methanol production from CO_2_ compared to that of other systems [50]. Moreover, biochemical approaches involving the introduction of conductive polyaniline hydrogels and nanobiocatalysts, which are graphene-immobilized enzymes, have increased CO_2_ conversion efficiency [51, 52].

Studies on producing materials other than methanol from CO_2_ have also been conducted. A novel CA from *Corynebacterium flavescens* in cow saliva was isolated, which produced up to 45 mg CaCO_3_/mg protein from CO_2_ through the optimization of the reaction parameters [53]. Another dehydrogenase involved in the rTCA cycle, isocitrate dehydrogenase from *Chlorobium limicola*, which can assimilate CO_2_ to 2-oxoglutarate, was characterized [54]. In addition to dehydrogenases for CO_2_ fixation, various oxidoreductases, such as pyruvate:ferredoxin oxidoreductase (PFOR), oxalate oxidoreductase (OOR), 2-oxoglutarate:ferredoxin oxidoreductase (OGOR), and other 2-oxoacid:ferredoxin oxidoreductases (OFORs), whose reactions are mediated by Fd as the electron mediator, have been explored. The function of CO_2_ fixation in these OFORs has been identified and analyzed based on model structures [55-57]. Efforts to discover highly active CO_2_ sequestrating enzymes are ongoing.

## Redox Cofactors and Cofactor Recycling

Not only enzymes but also redox cofactors that supply reducing power and energy play an important role in biological CO_2_ assimilation [58, 59]. Even for enzymes with reversible activity, cases exist in which one direction is more dominant than the other in general; notably, C1 fixation/reduction bias is more challenging than the reverse reaction. To overcome the thermodynamic barriers between substances, most carbon-fixing enzymes must receive electrons, either directly or through cofactors, which provide the driving force to reduce the C1 molecule. Therefore, the enzymes directly involved in carbon conversion are key elements, and the cofactors that promote enzymatic reactions are also critical to the overall reaction. A redox cofactor is essential for redox equivalence in terms of the electron carriers or mediators in efficient CO_2_ assimilation reactions. It takes and provides an electron or energy to other proteins depending on the driving force. The biological and chemical redox cofactors and their potentials are listed in Table 2. Some representative biological electron cofactors are NAD(P)^+^/NAD(P)H and Fd; ring-form materials, such as pyridine, quinone, and aniline, are used as chemical redox cofactors [60]. Among the chemical redox cofactors, viologen derived from 4,4’-bipyridine is widely used as a chemical electron mediator [61]. For example, CO_2_ is converted into carbon monoxide and formic acid at reduction potentials of –596 and –417 mV, respectively. To promote CO_2_ conversion, the redox cofactors with lower potential values than the –596 and –417 mV, including bipyridines, EcFd, and others, as listed in Table 2, can be applied to the CO_2_ reduction reaction [62]. These cofactors work as electron donors or acceptors, and the reaction can be sustained by regenerating cofactors. Cofactor regeneration is a stable and sustainable method for CO_2_ conversion that is cost-effective and highly productive [50]. Hence, the oxidized form of cofactors into the reduced form following C1 fixation must be reproduced for them to continuously act in cofactor-dependent enzymatic reactions. The CO_2_-to-methanol pathway, which is a representative C1 conversion pathway, usually requires NADH as a cofactor for C1 reduction. In each step of hydrogenation, C1 substances are reduced using the reducing power generated by NADH oxidation. NADH is regenerated while converting glutamate into 2-oxoglutarate by applying glutamate dehydrogenase to recycle NAD^+^ generated in C1 reduction [63-65]. In addition, other enzymes, such as glucose dehydrogenase and xylose dehydrogenase, that use NAD/NADH can also be used as recycling cofactors [66]. Other dehydrogenases, such as glycine dehydrogenase (GlyDH) and phosphite dehydrogenase (PTDH), catalyze glycine to glyoxylate and phosphite to phosphate for NADH regeneration [50, 67]. Moreover, these cofactor regeneration enzymes, PTDH and GlyDH, have optimal activities at neutral and basic pH, respectively [67]. This means that the cofactor regeneration system can be efficiently controlled according to pH.

Another breakthrough in cofactor regeneration was the application of a hybrid system with biological or chemical materials and electrochemicals [51, 68]. The use of electric power to replenish redox cofactors or provide a direct electron supply to biocatalysts is a robust and efficient approach. In the study of carbon conversion with cofactor regeneration via electrodes, diverse materials, both biological and chemical, can act as electron carriers; some systems that do not require an electron carrier can directly transfer electrons to biocatalysts. Regenerating NAD+ through an electrode with a continuous supply of electrons and conjugating FDH to a polydopamine-based bioelectrode film called PDA leads to the effective reduction of CO_2_ into formate [69]. Similar to the FDH-PDA bioelectrochemical method, an FDH-polyaniline hydrogel hybrid electrode was applied for effective CO_2_ reduction to provide a steady electron supply [51].

Studies into chemical redox polymers, such as cobaltocene-poly(allylamine), which function as electron mediators, have led to successful CO_2_ reduction through a continuous supply of electrons [70]. In the case of NAD-independent FDH, a sufficient amount of electrons provided by the electrode is transferred to the active site of FDH via iron–sulfur clusters to reduce CO_2_ [71]. Chemical materials, such as cobaltocene/cobaltocenium, were also used as electron mediators and recycled by electrodes to produce H_2_, CH_4_, C_2_H_4_, and C_3_H_6_ from H^+^ and CO_2_ [72]. In another study, for a chemical electron mediator, TiO2 was applied to carbon monoxide dehydrogenase. CO_2_ photoreduction was achieved by introducing silver nanoclusters, an electron-generating system that acts as a photosensitizer and an electron donor [73].

## Conclusions and Perspectives

Biological and biomimicked CO_2_ conversion is an important and promising field in terms of reducing GHGs and generating value-added materials from CO_2_ or methane. Until the discovery of a novel reductive glycine pathway in 2020, six CO_2_ fixation pathways were known from photoautotrophic and chemoautotrophic microorganisms. As such, more pathways to CO_2_ fixation metabolism may exist. Therefore, efforts to identify new strains hold promise for discovering novel pathways that can use C1. This will lead to the possibility of discovering enzymes with enhanced C1 reduction activity or finding novel C1 fixation pathway enzymes and cofactors that can convert C1. However, issues such as the energy difference between the substrates and products remain challenging to overcome in the quest for efficient carbon assimilation. To overcome these obstacles, we need to not only intensively study the native enzymes directly involved in C1 assimilation but also improve their C1 reduction activity and sustainability through mutant studies using synthetic biology techniques. Additionally, we must build heterologous detour pathways, optimize reaction conditions, and conduct studies on cofactors that help the carbon utilization reaction. Furthermore, convergence studies must be conducted between biology and other fields, such as electrochemistry and nanomaterials, from various perspectives to increase the efficiency of C1 reduction. Biological C1 conversion shows promise as a sustainable and efficient method for converting CO_2_ into valuable chemicals; however, further research and development are necessary for its widespread use. Therefore, the field of biological C1 assimilation has considerable potential in various application fields, and continuous research in this field will considerably contribute to fulfilling the global goal of net-zero carbon emissions by reducing GHG emissions and improving carbon resource recycling.

## Figures and Tables

**Table 1:** Natural CO_2_ assimilation pathways.

Name of pathway	Enzymes (EC Number)	CO_2_ fixation reaction	Simplified reaction	Cofactor requirements	Ref
Calvin-Benson-Bassham cycle (CBB)	Rubisco (4.1.1.39)	Ribulose-1,5-bisphosphate + CO_2_ → 3-Phospho glycerate	C5 + C1 → 2C3	-	[17]
Wood-Ljungdahl pathway (WLP)	Formate dehydrogenase (1.17.1.9)	CO_2_ + NADPH → Formate	C1 → C1	NADPH	[18]
	Carbon monoxide dehydrogeanse (1.2.7.4)	CO_2_ + Fd_red_ → CO	C1 → C1	reduced Fd	
reductive glycine pathway (rGlyP)/ Glycine cleavage/ synthase system (GCS)	Aminomethyltransferase (2.1.2.10)	5,10-methylene-THF + NH_3_ + CO_2_ + NADH → Glycine + THF + NAD^+^	One carbon unit + C1 → C2	NADH	[74]
	Glycine dehydrogenase (1.4.4.2)				
	Dihydrolipoyl dehydrogenase (1.8.1.4)				
3-Hydroxypropionate bicycle (3HP)	Acetyl-CoA carboxylase (6.4.1.2)	Acetyl-CoA + HCO_3_^-^+ ATP → Malonyl-CoA + ADP	C2-CoA + C1 → C3-CoA	ATP	[25]
	Propionyl-CoA carboxylase (6.4.1.3)	Propionyl-CoA + HCO_3_^-^+ ATP → (s)-Methylmalonyl-CoA + ADP	C3-CoA + C1 → C4-CoA	ATP	
3-Hydroxypropionate/ 4-Hydroxybutyrate cycle (3HP/4HB)	Acetyl-CoA carboxylase (6.4.1.2)	Acetyl-CoA + HCO_3_^-^+ ATP → Malonyl-CoA + ADP	C2-CoA + C1 → C3-CoA	ATP	[28]
	Propionyl-CoA carboxylase (6.4.1.3)	Propionyl-CoA + HCO_3_^-^+ ATP → (s)-Methylmalonyl-CoA + ADP	C3-CoA + C1 → C4-CoA	ATP	
Dicarboxylate/4-Hydroxybutyrate cycle (DC/4HB)	Pyruvate synthase (1.2.7.1)	Acetyl-CoA + CO_2_ + Fd_red_ → Pyruvate + Fd_oxi_	C2-CoA + C1 → C3	Reduced Fd	[26]
	Phosphoenolpyruvate carboxylase (4.1.1.31)	Phosphoenolpyruvate + HCO_3_^-^→ Oxaloacetate	C3 + C1 → C4	-	
reverse Tricarboxylic acid cycle (rTCA)	2-Oxoglutarate oxidoreductase (1.2.7.3)	Succinyl-CoA + CO_2_ + Fd_red_ → 2-Oxoglutarate + Fd_oxi_	C4 + C1 → C5	Reduced Fd	[29]
	Isocitrate dehydrogenase (6.4.1.7)	2-Oxoglutarate + CO_2_ + NAD(P)H → Isocitrate + NAD(P)^+^	C5 + C1 → C6	NAD(P)H	

**Table 2:** Representative biological and chemical redox cofactors.

Group	Type of electron carrier	PDB ID	Prosthetic groups	Redox potential (mV)	Condition	Ref
Redox couples	NAD^+^/NADH			-320	pH 7.0	[75]
	NADP^+^/NADPH			-320	pH 7.0	[75]
	FMN/FMNH_2_			-380	pH 7.0	[76]
	FAD/FADH_2_			-208	pH 7.0, 25°C	[77]
Ferredoxins	AlvinFd	1BLU	2[4Fe-4S]	-467, -640	pH 7.0	[78]
	AvFd	6FD1, 7FD1	[3Fe-4S], [4Fe-4S]	-420, -650	pH 7.0, 0°C	[78] [79]
	BpFd	ND	1[4Fe-4S]	-390	pH 6.0 to 7.5	[80]
	BtFdI/II	1IQZ/1IR0	1[4Fe-4S]	ND	ND	[81]
	CaFd	1FCA, 2FDN	2[4Fe-4S]	ND	ND	[81]
	ClFd	ND	2[4Fe-4S]	<-500	ND	[58]
	CpFd	1CLF	2[4Fe-4S]	-420	pH 7.0	[78]
	CtFdI/II	ND	2[4Fe-4S]	-514/-584	pH 7.5, at 25°C	[82]
	CvFd	1BLU	2[4Fe-4S]	-461, -653	pH 7.5, at 25°C	[83]
	DaFdI	1FXR, 1DAX	1[4Fe-4S]	-385	pH 7.0, at 23°C	[81]
	EcFd	2ZVS	2[4Fe-4S]	-418, -675	pH 7.0	[84]
	EhFd	ND	2[4Fe-4S]	-333	pH 7.0	[84]
	HtFd1	7M1N	1[4Fe-4S]	-485	pH 7.0, at 23°C	[59]
	MmFd1/2/3	ND	2[4Fe-4S]	-485, -635/-520/-233, -380	pH 7.0	[57]
	MtFd	ND	2[4Fe-4S]	-454, -487	pH 7.6	[85]
	PaFd	2FGO	2[4Fe-4S]	-475, -655	pH 7.0	[81]
	SaFd	ND	[3Fe-4S], [4Fe-4S]	-275, -529	pH 6.4, 0°C	[86]
	TaFd	1RGV	2[4Fe-4S]	-431, -587	pH 7.0, 0°C	[87]
	TmFd	1VJW, 1ROF	2[4Fe-4S]	-420	pH 7.0, 0°C	[81]
	TtFd	1H98	[3Fe-4S], [4Fe-4S]	ND	ND	[81]
Pyridine	Benzyl viologen			-578.2/-745.4	pH 7.4, 25°C	[88]
	Ethyl viologen			-701.5/-992.3	pH 7.4, 25°C	
	Methyl viologen			-697.5/-1029.5	pH 7.4, 25°C	
	1,1’-Diheptyl-4,4’-bipyridinium			-626.2/-786.2	pH 7.4, 25°C	
	1,1’-Diphenyl-4,4’-bipyridinium			-457.5	pH 7.4, 25°C	
	4,4'-Dipyridyl			-1080	pH 7.4, 25°C	
	1-Heptyl-4-(4-pyridyl) pyridinium			-949	pH 7.4, 25°C	
	2-Hydroxy-1,4-naphthoquinone			-535.4	pH 7.4, 25°C	
Quinone	2-Methyl-1,4-Naphthoquinone			-411.7	pH 7.4, 25°C	

Abbreviations: Alvin: *Allochromatium vinosum*; Av: *Azotobacter vinelandii*; Bp: *Bacillus polymyxa*; Bt: *Bacillus thermoproteolyticus*; Ca: *Clostridium acidurici*; Cl: *Chlorobium limicola*; Cp: *Clostridium pasteurianum*; Ct: *Chlorobium tepidum*; Cv: *Chromatium vinosum*; Da: *Desulfovibrio africanus*; Ec: *Escherichia coli*; Eh: *Entamoeba histolytica*; Ht: *Hydrogenobacter thermophiles*; Mm: *Magnetococcus marinus*; Mt: *Moorella thermoacetica*; Pa: *Pseudomonas aeruginosa*; Sa: *Sulfolobus acidocaldarius*; Ta: *Thauera aromatica*; Tm: *Thermotoga maritima*; Tt: *Thermus thermophiles*; ND: not determined.
